# REMEMBER AND REMINISCE: CONNECTING OUR SCIENCE TO PEOPLE’S LIVES

**DOI:** 10.1093/geroni/igz038.1125

**Published:** 2019-11-08

**Authors:** Joseph E Gaugler, Robyn Birkeland, Tamara L Statz, Rachel Zmora, Colleen M Peterson, Hayley R McCarron, Nancy Kazlauckas

**Affiliations:** 1 University of Minnesota - School of Public Health, Division of Health Policy and Management, Minneapolis, Minnesota, United States; 2 University of Minnesota, Minneapolis, Minnesota, United States; 3 University of Minnesota - School of Public Health, Division of Epidemiology and Community Health, Minneapolis, Minnesota, United States; 4 University of Minnesota School of Public Health, Minneapolis, Minnesota, United States; 5 Independent Quilting Artist, Sauk Centre, Minnesota, United States

## Abstract

A driving paradigm of science, including much of the discipline of gerontology, remains post-positivism. However, the post-positivistic emphasis on experimental control, elimination of “bias,” and internal validity may create a chasm between the science and scholarship we conduct and the lives of those who engage with us in such endeavors. The purpose of this presentation (a quilt provided to the authors by an individual who completed one of our studies evaluating the efficacy of a psychosocial support program for family members of relatives living in residential long-term care and with memory loss) is to offer an opportunity for scholars and others to reflect on the connections and partnerships we establish with those who volunteer to engage in our research initiatives. In particular, the aim of this presentation is to stimulate viewers to consider how we grow our relationships with those who volunteer in our research by creating true partnerships that outlast a single project. With the emergence of participatory research methodologies, person-centered care, and social media technologies, the importance of building relationships and partnerships with communities, families, and individuals in a more equitable manner is pressing. The use of the “Remember and Reminisce” quilt as well as other materials donated by individuals to dementia care researchers provide vivid, tactile reminders from those who engage with our science that our obligations to understanding their experiences must assume a broader, longer-term perspective than the boundaries that often dictate the life-cycle of a standard project/study.

